# Cyclic voles and shrews and non-cyclic mice in a marginal grassland within European temperate forest

**DOI:** 10.1007/s13364-012-0072-2

**Published:** 2012-02-14

**Authors:** K. Zub, B. Jędrzejewska, W. Jędrzejewski, K. A. Bartoń

**Affiliations:** Mammal Research Institute, Polish Academy of Sciences, 17-230 Białowieża, Poland

**Keywords:** Autoregressive model, Climatic factors, Common shrew, Population cycles, Root vole, Spectral analysis, Striped field mouse

## Abstract

Cyclic population dynamics of small mammals are not restricted to the boreal and arctic zones of Eurasia and North America, but long-term data series from lower latitudes are still less common. We demonstrated here the presence of periodic oscillations in small mammal populations in eastern Poland using 22-year (1986–2007) trapping data from marginal meadow and river valley grasslands located in the extensive temperate woodland of Białowieża Primeval Forest. The two most common species inhabiting meadows and river valleys, root vole *Microtus oeconomus* and common shrew *Sorex araneus*, exhibited synchronous periodic changes, characterised by a 3-year time lag as indicated by an autocorrelation function. Moreover, the cycles of these two species were synchronous within both habitats. Population dynamics of the striped field mouse *Apodemus agrarius* was not cyclic. However, this species regularly reached maximum density 1 year before the synchronized peak of root voles and common shrews, which may suggest the existence of interspecific competition. Dynamics of all three species was dominated by direct density-dependent process, whereas delayed density dependent feedback was significant only in the root vole and common shrew. Climatic factors acting in winter and spring (affecting mainly survival and initial reproduction rates) were more important than those acting in summer and autumn and affected significantly only the common shrew. High temperatures in winter and spring had positive effects on autumn-to-autumn changes in abundance of this species, whereas deep snow in combination with high rainfall in spring negatively affected population increase rates in common shrew.

## Introduction

Periodic oscillations of small mammal numbers have fascinated biologists throughout several decades; however, there is still considerable controversy about the mechanisms underlying this process (Elton 1924; Korpimäki and Krebs 1996). At least 20 hypotheses have been formulated, invoking the effect of specialist predators, food shortage, or intrinsic factors (Batzli 1992; Smith et al. 2006). The slow progress in piecing together the puzzle of rodent population cycles stems mainly from the lack of appropriate data because ecological time series are often too short to demonstrate statistical significance (Berryman 2002).

Another problem is a definition of the “population cycle.” According to Berryman (2002), it is “an oscillation in population number or density that has an obviously regular period of three or more years.” Here, we adopt this definition of population cycles. Some researches narrow the definition of cycles to oscillations of high amplitude accompanied by large spatial and close temporal synchrony with other species (Korpimäki et al. 2004). Other features of cyclic populations include a summer crash of rodent abundance and prolonged low-density phase (Korpimäki and Krebs 1996; Boonstra et al. 1998; Gilg 2002). If we are to accept this definition, the term “cycles” would apply only to periodic oscillations of voles in the boreal zone of Eurasia, lemmings in the arctic zones of Eurasia and North America, snowshoe hares in the boreal zone of North America, and house mice in southeastern Australia (Boonstra et al. 1998; Erlinge et al. 2000; Hanski et al. 1991; Klemola et al. 2003; Krebs et al. 1995; Saitoh 1987; Sinclair et al. 1990; Singleton et al. 2001; Stenseth 1999).

Periodic fluctuations of rodent species are, however, observed also in temperate zones, and they suit very well the definition of cyclic populations given by Berryman (2002) (Jędrzejewski and Jędrzejewska 1996; Lambin et al. 2000; Tkadlec and Stenseth 2001). These studies are often treated as of marginal significance due to low amplitude of rodent density changes and small scale of this phenomenon (Korpimäki et al. 2004). On the other hand, in seeking for a more universal explanation of rodent cycles, one cannot ignore and marginalize long-term data collected outside the boreal and arctic zones.

In Europe, several distinct geographical gradients of vole cycles have been found (Jędrzejewski and Jędrzejewska 1996; Tkadlec and Stenseth 2001), of which the best known, the Fennoscandian gradient, is characterized by a decreasing amplitude and length of oscillation period from north to south (Hanski et al. 1991). In Fennoscandia, vole cycles vanish below 60° N, yet there are no premises to suppose that they do not exist in Central or Southern Europe (Jędrzejewski and Jędrzejewska 1996).

The aim of our study was to analyse dynamics of cyclic small mammal populations in marginal open habitats located within extensive woodlands of Białowieża Primeval Forest (BPF, Central Europe). Populations of small mammals in BPF inhabit seminatural sedge meadows in the river valleys and abandoned moist and dry meadows—environments characterized by low human impact. Previous studies concerning small mammal cycles in Central Europe focused largely on the common vole *Microtus arvalis* and were conducted in extensive, cultivated landscapes (Tkadlec and Stenseth 2001). Cycles of field voles *Microtus agrestis* in northern England were also studied in a transformed landscape, on isolated clear-cuts within the forest (Lambin et al. 2000). In our study area, the communities of cyclic small mammals inhabit open habitats which compose natural or seminatural part of a bigger ecosystem, the lowland primeval deciduous and mixed forest. These conditions offer us the possibility to consider more complex natural interactions between various species of small mammals, their habitats and their predators (comp. Jędrzejewska and Jędrzejewski 1998). In Europe, population cycles in small mammals have been vanishing during the past two decades and climate change is most often invoked as the main cause of this process (Ims et al. 2008). In this paper, we analysed 22-year-long trapping data of the most common species and related them to climatic conditions, which could modify the response of small mammal populations to density-dependent factors. We focused on three climatic variables—ambient temperature, precipitation and snow cover depth, which directly affect energy expenditure in small mammals and their survival (Hayes and O'Connor 1999). The climatic factors may act also indirectly, e.g. mild winter conditions can lead to accumulation of ice, limiting access to food resources (Korslund and Steen 2006).

## Materials and methods

### Study area

**Fig. 1:**
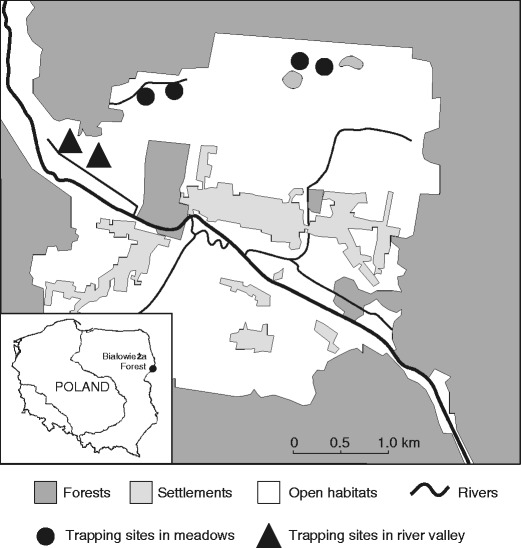
Location of small mammal trapping sites in marginal open habitat (meadow and river valley) in Białowieża Primeval Forest, E Poland

We conducted our study in the central part of the Białowieża Primeval Forest (23.86° E, 52.70° N), E Poland. Within the study area, located on the Białowieża Glade (13.5 km^2^), we selected two habitat types in open areas. The first type of habitat (hereafter referred to as “meadows”) included four trapping sites and was located north of Białowieża village in meadows and unmanaged grasslands surrounded by forest, arable land and settlements (Fig. 1). Vegetation types occurring in this plot were fresh meadow and pasture communities, including abandoned ones (*Agropyro-Rumicion*, *Arrhenatherion*), as well as dry meadows and unused grassland (*Calluna-Nardetea*, *Sedo-Scleranthetea*). The second habitat type (hereafter referred to as “river valley”) included two trapping sites along the Narewka river valley (Fig. 1). This habitat type has been gradually abandoned and has become a mosaic of diverse plant communities: reeds (*Phragmitetea*), wet and moist meadows (*Molinio-Arrhenatherethea*), mires (*Scheuchzerio-Caricetea*), fresh meadows, dry meadows and unused grassland, willow and willow-alder brushwood, and alder-wood (Falińska 2003). In the past, both study sites were mown regularly, but since the 1970s, only small patches were used. The two habitat types were characterised by distinct water regimes due to persistence of floodwater in spring and autumn in the river valley. Both habitat types were adjacent to the Białowieża Primeval Forest, thus rodent communities in these habitats are influenced by the abundance of predators and their prey in woodlands (Fig. 1).

### Rodent trapping

Small mammals were trapped at the end of September or beginning of October, from 1986 to 2007. Trapping session at each site lasted for 6 days on average (occasionally 5 or 8 days). Each trapping site consisted of four live traps (baited with oats), two metal cones (pitfall traps dug into the ground), and, until year 2000, additionally four snap traps (baited with oil and parsley). In 2001, we ceased using snap traps due to change in the ethical regulations. Thus, we used 14 traps until year 2000 and ten traps per site from 2001 to 2007.

In the meadows, the four trapping sites were located about 2 km apart, and in the river valley, two trapping sites were 500 m apart (Fig. 1). Traps were checked once a day, between 0800 and 1100 hours. To avoid repeated captures, all animals were collected and kept in the laboratory until the end of trapping session and were released in the place of capture on the last trapping day. In the laboratory, we identified the species by their external features. In total, we captured 1,212 specimens, 729 in the meadows and 483 in the river valley (Table 1). On average, during one trapping session (six trapping sites), we captured 55.1 individuals (SD = 26.6; range, 22–111 individuals).

**Table 1:** Structure of the small mammal community in the open habitats of Białowieża Primeval Forest

Species	Percentage of all small mammals trapped	
	Meadows	River valley
**Root vole** ***Microtus oeconomus***	**38.8**	**39.5**
Common vole *Microtus arvalis*	6.9	1.9
Field vole *Microtus agrestis*	3.3	0.2
Bank vole *Myodes glareolus*	4.0	11.0
**Field striped mouse** ***Apodemus agrarius***	**13.9**	**5.8**
Yellow-necked mouse *Apodemus flavicollis*	2.7	1.0
Harvest mouse *Micromys minutus*	3.2	3.7
Northern birch mouse *Sicista betulina*	0.1	–
**Common shrew** ***Sorex araneus***	**21.9**	**27.1**
Pygmy shrew *Sorex minutus*	4.7	5.6
Laxmann's shrew *Sorex caecutiens*	0.1	–
Water shrew *Neomys fodiens*	0.4	2.9
Mediterranean water shrew *Neomys anomalus*	–	1.2
Total *N* individuals	729	483

Share of species in the community was calculated based on all specimens trapped between 1986 and 2007. Dominant species are marked in boldface

### Weather data

Climatic data were collected at the meteorological station located in Białowieża village, about 1 km from the trapping sites. The climate of BPF is transitional between Atlantic and continental types with clearly marked cold and warm seasons. The mean annual temperature in 1986–2007 was 8.0°C (range of mean daily temperatures, −24.7 to 28.8°C). The coldest month was January (mean daily temperature, −2.7°C), and the warmest was July (19.7°C). Snow cover persisted for an average of 78 days per year with a mean depth of 13.0 cm. Yearly precipitation averaged 600 mm. Most of the rainfall occurred in summer (June–August, 203.3 mm, with maximum in July, 74.7 mm), but spring (March–May) and autumn (September–November) were characterized by average precipitation (139.6 and 140.1 mm, respectively). In winter (December–February) precipitation was lowest (117.0 mm, with minimum in February, 35.5 mm).

### Statistical analyses

We calculated mean abundance indices (*N* individuals captured per 100 trap nights), their amplitude (average difference between the highest and lowest ln-transformed abundance using a 5-year moving window) and *s*-index (cyclicity index-standard deviance of log_10_ abundances, Henttonen et al. 1985). Further analyses were performed on autumn-to-autumn differences of ln(*x* + 1) transformed abundance indices (per capita population growth rate) rather than on the raw log-abundance index for two reasons. Firstly, the process of changes in small mammal number is multiplicative and as such should be considered on a logarithmic scale (Bjørnstad et al. 1996; Royama 1992). Secondly, the data in the time series analyses must be stationary, i.e. mean, variance and covariance of the data series cannot depend on the time period. Calculating abundance changes based on a logarithmic rather than a raw scale allowed us to comply with this assumption (augmented Dickey–Fuller stationarity test). Prior to analyses, we detrended the abundance indexes by subtracting the species-specific mean abundance index. We analysed the data collected from the meadows and river valley separately due to differences in the composition of small mammal communities and potentially different climatic effects on the population dynamics in the two habitats.

To explore temporal patterns, we utilised autocorrelation function (ACF) and partial rate correlation function (PRCF) separately for different species and habitats. To assess relationships between the abundance dynamics, we also used a cross correlation function (CCF), which allowed us to locate a significant time lag between the species. We estimated 95% confidence intervals for the coefficients of autocorrelation or partial rate correlation function at lag *k*, by comparing them with the critical values ±1.96/*n* ^½^, where *n* is the number of observations (Bartlett’s significance band).

We identified the relative contribution of first- and second-order negative feedbacks (C1 and C2) by fitting the Gompertz function of form:

1 $$ {R_t} = A + {\text{C1}}\,{X_t}_{ - {1}} + {\text{C2 }}{X_t}_{ - {2}} + {e_t} $$

where *R*_*t*_ is (per capita growth rate, *R*_*t*_ = *X*_*t*_ − *X*_*t*−1_) and *X*_*t*_ is the natural logarithm of the abundance index (*N*) in autumn of year *t*. We used least squares linear model to fit the function. To test for relative importance of direct density-dependent effect, in addition to the above model, we fitted models with only 1 year lag (without the third term in Eq. 1).

We then inspected the PRCF (Berryman and Turchin 2001) plotted against time lags to identify whether the population dynamics was dominated by first- or second-order negative feedback (respectively, direct and delayed density dependence). In order to check if PRCF showed significant second-order signal, we made a visual inspection of *R*_*t*_ plotted against *N*_*t*−1_, to detect spurious second-order autocorrelation caused by strong nonlinearity in a first-order dynamic. In a first-order process, all data points should fall on or close to a continuous function, whereas in a second-order process, the data should form an elliptical orbit with no functional relationship (Berryman and Lima 2007).

To find possible discontinuity and examine the effect of the 2001 trapping design change (see section “Rodent trapping”) on periodicity of dynamics of small mammal populations we compared models without and with separation of these two periods using *F* test. The latter model included the model described by Eq. 1 nested within each period.

We also attempted to identify climatic factors, which could modify responses of small mammal populations to density-dependent processes. In our study area, small mammals reproduce from March till September, and we performed trapping after breeding season when their density was highest. The most critical period is late winter and spring, when unfavorable weather conditions (e.g. low ambient temperatures, high precipitation causing flooding) may affect survival and substantially reduce density of small mammals (Wijnhoven et al. 2005). In winter, the negative effect of low temperatures can be mitigated by deep snow cover, which additionally serves as protection against predators. On the other hand, the combination of snow cover with high temperatures leads to accumulation of ice, limiting access to food resources (Korslund and Steen 2006). During warm periods, ambient temperatures and precipitation may affect availability of food resources and indirectly influence growth of small mammal populations (Jędrzejewski and Jędrzejewska 1996).

First, we examined a large set of climatic factors (mean, minimum and maximum values of ambient temperature, precipitation, snow depth, and number of days with snow cover) and interactions between them. Climatic variables were calculated as means, separately for each of the four seasons (winter, December to February; spring, March to May; summer, June to August and autumn, September to November). Seasons were defined according to prevailing weather conditions and the reproductive status of small mammals. Those preliminary analyses revealed that only mean ambient temperature, precipitation and snow depth significantly affected the population dynamics of small mammals. Thus, in final analyses, we used only these three climatic variables.

Climatic variables and interactions between them were included in the model described by Eq. 1 with lag-order determined by PRCF function, with detrended log-abundance as a response variable. We constructed a set of candidate models including effect of density-dependent feedback (with lag-order determined by PRCF function), mean ambient temperature, mean snow cover and mean precipitation, and used model selection procedure (Burnham and Anderson 2002) with models ranked by AIC_c_ (corrected Akaike’s Information Criterion). To compare models, we used delta AIC_c_, a measure of each model relative to the best model, and as a rule of thumb all models Δ_*i*_ < 2 were regarded as equally supported (Burnham and Anderson 2002). When more than one model was supported, we used the confidence set of candidate models, which included models with Akaike weights that were within 10% of the value of the highest ranked model. Finally, we estimated average values of parameter along with confidence intervals, calculated using unconditional standard errors (Burnham and Anderson 2002). To evaluate the effect of climatic variables on population dynamics of small mammals, we compared models including only effect of density-dependent feedback (with lag-order determined by PRCF function) with models, which included all climatic factors, determined for each species by the above-described procedure. For the statistical analysis, we used two packages: the Population Analysis System PAS (Berryman 1999) and R (R Development Core Team 2011).

## Results

From 1986 to 2007, we captured small mammals of 13 species (eight rodents and five insectivores) (Table 1). In both habitats, the two most abundant species were the root vole *Microtus oeconomus,* and the common shrew *Sorex araneus*. In the meadows, the third most numerous species was the striped field mouse *Apodemus agrarius*, but in the river valley, it was the bank vole *Myodes glareolus*. The pooled proportion of the remaining species in the community was below 10% (Table 1). For this reason, in the analysis we focused on the three most common species (Table 1). These species have been captured each year in both habitats, whereas captures of the remaining species were distributed unevenly among years and habitats.

Since 2001, we observed an increase in the abundance indices of small mammals, i.e. for the same number of captured animals indices were higher, this was because of the lower number of traps due to the cessation of snap trapping. This increase was proportional to the change of total number of trap nights during one trapping session. In the meadows, the mean number of trap nights (±SD) decreased by a factor of 1.9, from 242.7 (±23.7) to 130.3 (±12.8) and, at the same time, the mean indices for all small mammals changed by 1.8 times, from 14.3 (±8.5) to 25.4 (±9.8) individuals per 100 trap nights. In the river valley, the change in number of trap nights was similar (by 1.9 times), and the mean abundance indices increased by 1.7 times, from 18.9 (±11.3) to 31.9 (±11.3). This, however, did not affect the periodicity of dynamics of small mammal populations.

Over the entire study period, the maximum small mammal abundance index, calculated for each trapping session separately, was 42.5 individuals per 100 trap nights in the meadows and 45.8 in the river valley. It means that each night at least half of the traps were unoccupied, and the index was affected only by the total number of traps used.

Thus, for analyses of long-term changes, we assumed that every year the number of trap nights was the same. Using this conservative reasoning, we observed significant differences in abundance of some species between the first (1986–2000) and the second part (2001–2007) of the study period. Analysing the effect of the study period on the abundance indices, we included in the models a factor indicating the first or second part of the study period as a fixed effect and the habitat as a random effect.

The mean abundance index in the second period increased in field mouse (ANOVA, *F*_1,41_ = 8.22, *p* = 0.007) and common shrew (*F*_1,41_ = 7.60, *p* = 0.008), but did not change in root vole (*F*_1,41_ = 0.18, *p* = 0.66). We found a significant effect of the study period in yellow-necked mouse (*F*_1,41_ = 10.90, *p* = 0.002), which was also more abundant in the second period but not in the bank vole (*F*_1,41_ = 1.46, *p* = 0.23) or harvest mouse (*F*_1,41_ = 0.54, *p* = 0.46). The effect of habitat was not significant for any species, except the bank vole (*F*_1,41_ = 6.72, *p* = 0.01) and water shrew (*F*_1,41_ = 7.87, *p* = 0.008), which were more abundant in the river valley. The comparison of models with separation of the two trapping periods did not show significant differences for any species or habitats (all *p* ≫ 0.1).

**Fig. 2:**
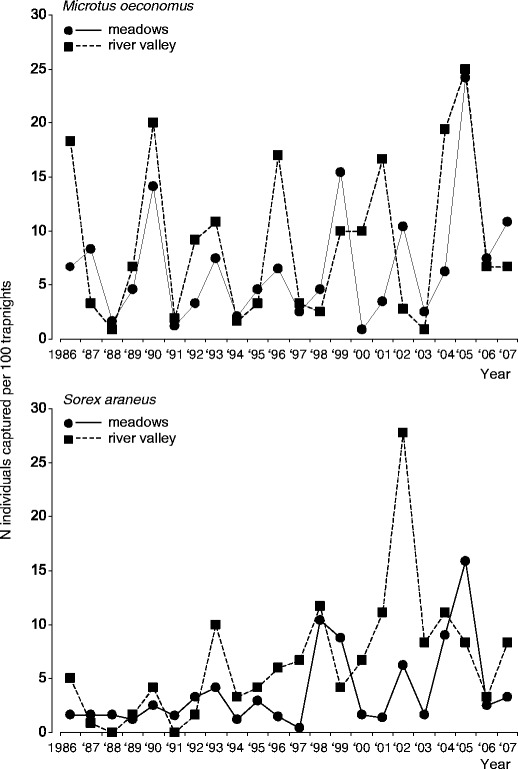
Population dynamics of root vole *M. oeconomus* and common shrew *S. araneus* in open habitats of BPF, in autumn (September–October) in years 1986–2007

**Fig. 3:**
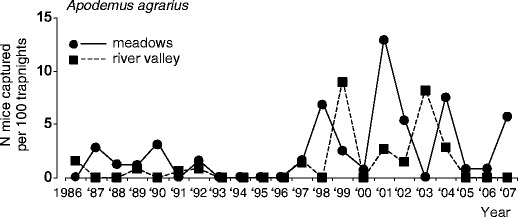
Population dynamics of field mouse *A. agrarius* in meadows and river valley in BPF in autumn (September–October), 1986–2007

All three most common species (root vole, common shrew, and field mouse) underwent oscillations characterized by moderate amplitudes, ranging from 1.51 to 2.93 inds/100 TN and a low *s*-index, ranging from 0.32 to 0.51 (Figs. 2 and 3; Table 2). Only for the root voles inhabiting the river valley, the *s*-index of 0.51 marginally exceeded an arbitrary threshold (0.5) suggested by Henttonen et al. (1985) as useful for distinguishing cyclic from non-cyclic populations. Oscillations of two dominating species—root vole and common shrew—were very regular. In the meadows, the time lag of periodic changes, indicated by ACF was 3 or 6 years, but it was significant only for the root vole and common shrew (Table 2). In the river valley, the time lag was 3 or 4 years, but not significant for any species. The oscillations of field mouse abundance in the river valley were characterized by a shorter and not significant time lag (Table 2).

**Table 2:** Descriptive statistics for oscillations of small mammal populations in open habitats of BPF, 1986–2007

Species	Mean abundance (*N*/100 TN)	Amplitude	*s*-index	Lag (in years) of ACF (coefficient)
Meadows				
*Microtus oeconomus*	6.78	2.23	0.37	3 (0.63), 6 (0.43)
*Sorex araneus*	3.85	1.94	0.38	3 (0.20), 6 (0.42)
*Apodemus agrarius*	2.56	1.83	0.36	3 (0.34)
River valley				
*Microtus oeconomus*	8.91	2.93	0.51	3 (0.13), 4 (0.10), 6 (0.15)
*Sorex araneus*	6.60	1.86	0.47	3 (0.17), 4 (0.09)
*Apodemus agrarius*	1.37	1.51	0.32	2 (0.23)

Mean abundance index: *N* individuals captured per 100 trap nights (N/100 TN) in autumn (September–October). Significance level for ACF coefficients according to Bartlett’s test was 0.42

**Fig. 4:**
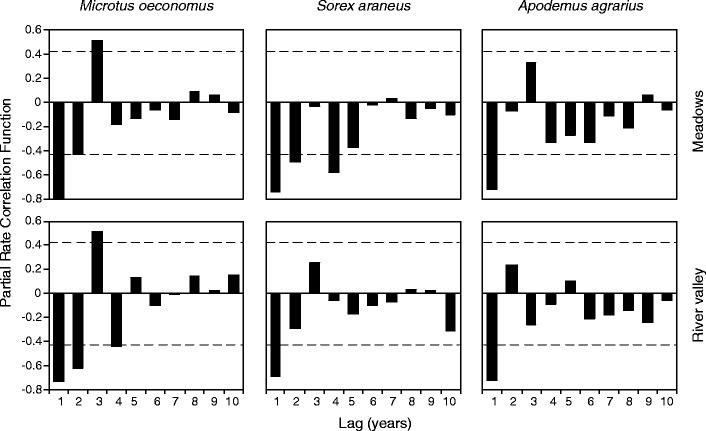
PRCF for the detrended time series of population dynamics of the three most common species of small mammals. The *dashed horizontal line* is Bartlett’s significance band

The partial rate correlation functions (PRCF) for all three species in two habitats (meadows and river valley) showed that the dynamics were dominated by first-order negative feedback (largest negative PRCF at lag 1, Fig. 4), whereas second-order negative feedback (largest negative PRCF at lag 2, Fig. 4) was significant only for the root vole and common shrew in the meadows and the root vole in the river valley.

Next, we used multiple regression to fit one-lag and two-lag logarithmic models to the data for three most common species (Table 3). Our analyses revealed that in all species, direct density-dependent feedback process explained over 50% of the variation in the data, while second-order negative feedback was involved mainly in the dynamics of root voles in the river valley (Table 3). We detected a significant second-order negative feedback also in the root vole and common shrew in the meadows, but not in the common shrew in the river valley (Table 3). The oscillations of the field mouse in both habitats were pure first-order process (Table 3). Visual inspection of the relationship between growth rate (*R*) and ln abundance in year *n* − 1 (*X*_*t*−1_) of the root vole and common shrew did not reveal spurious second-order autocorrelation caused by strong nonlinearity in a first-order dynamics.

**Table 3:** Parameter estimates of time-series models on the population growth rate (from autumn year *n* to autumn year *n* + 1) of the root vole, common shrew, and field striped mouse in meadows and the river valley

Coefficient/statistic	Ln *N*_*t*−1_	Ln *N*_*t*−2_	Ln *N*_*t*−1_ + Ln *N*_*t*−2_
*Microtus oeconomus* in meadows			
*A* (intercept)	5.20^a^	0.32	7.78^a^
C1 (slope_*t*−1_)	−1.27^a^	–	−1.44^a^
C2 (slope_*t*−2_)	–	−0.08	−0.46^c^
*r* ^2^	0.63^a^	0.03	0.74^a^
*r*_partial_^2^	0.74^a^	0.24^c^	–
*Sorex araneus* in meadows			
*A* (intercept)	4.06^b^	1.34	6.32^a^
C1 (slope_*t*−1_)	−1.11^b^	–	−1.18^a^
C2 (slope_*t*−2_)	–	−0.37	−0.54^c^
*r* ^2^	0.55^b^	0.06	0.67^a^
*r*_partial_^2^	0.65^a^	0.27^c^	–
*Apodemus agrarius* in meadows			
*A* (intercept)	3.42^a^	0.13	3.53^c^
C1 (slope_*t*−1_)	−1.06^a^	–	−1.03^a^
C2 (slope_*t*−2_)	–	−0.04	−0.07
*r* ^2^	0.53^a^	0.00	0.53^b^
*r*_partial_^2^	0.52^a^	0.01	–
*Microtus oeconomus* in river valley			
*A* (intercept)	4.56^a^	2.44^d^	7.63^a^
C1 (slope_*t*−1_)	−1.09^a^	–	−1.14^a^
C2 (slope_*t*−2_)	–	−0.57^d^	−0.67^a^
*r* ^2^	0.56^a^	0.17^d^	0.76^a^
*r*_partial_^2^	0.72^a^	0.49^a^	–
*Sorex araneus* in river valley			
*A* (intercept)	3.67^a^	1.56	4.86^b^
C1 (slope_*t*−1_)	−0.92^a^	–	−0.86^b^
C2 (slope_*t*−2_)	–	−0.38	−0.34
*r* ^2^	0.48^a^	0.08	0.50^c^
*r*_partial_^2^	0.46^b^	0.11	–
*Apodemus agrarius* in river valley			
*A* (intercept)	3.00^a^	−1.01	1.93
C1 (slope_*t*−1_)	−0.97^a^	–	−0.95^a^
C2 (slope_*t*−2_)	–	0.32	0.32
*r* ^2^	0.48^a^	0.05	0.53^b^
*r*_partial_^2^	0.50^a^	0.10	–

*Ln N*_*t−1*_, *Ln N*_*t−2*_ parameter estimates for model including only lag 1 or lag 2, respectively; *Ln N*_*t−1*_ *+ Ln N*_*t−2*_ parameter estimates for two-lag logarithmic model; *A* maximum per capita rate of change; *C1*, *C2* effect of density-dependent feedback with lag 1 and lag 2; *r* ^*2*^ amount of variation explained by density-dependent feedback; *r* ^*2*^_*partial*_ partial correlation

^a^Significance code, 0.001

^b^Significance code, 0.01

^c^Significance code, 0.05

^d^Significance code, 0.1

Population dynamics of the two dominant small mammal species, root vole and common shrew, were synchronised both between habitats and between species (significant lack of time-lag in CCF). Oscillations of field mouse abundance were not synchronised between habitats (Fig. 2), but in the meadows, the maximum abundance of this species in most cases fell 1 year before the peak of root vole (significant negative 1-year time-lag indicated by CCF).

We examined the effects of mean monthly temperature, rainfall and snow cover on autumn-to-autumn population changes of the studied populations. Selection procedure revealed that no single model were supported, and for all species, mean coefficients calculated for climatic factors were not significantly different from zero, except for the common shrew population inhabiting meadows (Table 4). The proportion of variation in population increase rates, explained by climatic factors, was much higher in the common shrew than in other species. In meadows, direct and delayed density-dependent feedback and climatic factors explained 92% of the variation in abundance of the common shrew, whereas in the river valley, direct density-dependent feedback and climatic factors explained 67% of the variation in abundance of this species (Table 4). The proportion of explained variation in abundance of the common shrew increased by 25% and 17%, in meadows and the river valley respectively, in comparison with the effect of density-dependent feedback only (Tables 3 and 4). In other species, proportion of explained variation in abundance increased only from 8% to 14%, and influence of climatic variables was insignificant or weakly significant (Table 4).

**Table 4:** Estimates of averaged model parameters with 95% confidence intervals (in parentheses) for the effect of climatic factors on population increase rate of small mammals

Climatic variable	Meadows			River valley		
	*M. oeconomus*	*S. araneus*	*A. agrarius*	*M. oeconomus*	*S. araneus*	*A. agrarius*
*T*_win_	0.04 (−0.08, 0.16)	0.20 (0.11, 0.28)	0.06 (−0.11, 0.23)	0.01 (−0.03, 0.05)	–	−0.05 (−0.20, 0.10)
*S*_win_	0.03 (−0.04, 0.11)	–	0.02 (−0.04, 0.07)	–	–	–
*P*_win_	–	–	–	0.18 (−0.42, 0.77)	–	–
*T*_spr_	−0.03 (−0.14, 0.09)	–	–	–	0.17 (−0.10, 0.45)	–
*S*_spr_	–	0.13 (0.01, 0.24)	−0.01 (−0.04, 0.02)	–	–	−0.01 (−0.05, 0.02)
*P*_spr_	–	0.29 (−0.04, 0.62)	–	−0.12 (−0.46, 0.21)	–	–
*P*_spr_ × S_spr_	–	−0.03 (−0.06, 0.00)	–	–	–	–
*T*_sum_	–	–	–	–	–	0.11 (−0.21, 0.43)
*T*_aut_	–	–	–	–	−0.18 (−0.47, 0.12)	–
*r* ^2^	0.85^a^	0.92^a^	0.67^a^	0.84^a^	0.67^a^	0.67^a^
*F*	3.37^b^	10.06^a^	2.30	2.26	5.30^b^	2.53^c^

*T* mean ambient temperature, *S* mean depth of snow cover, *P* mean precipitation, *spr* spring, *sum* summer, *aut* autumn, *win* winter, *r* ^*2*^ total amount of variation explained by direct and delayed density-dependent feedback and climatic factors (root vole in both habitats and common shrew in meadows) or by direct density-dependent feedback and climatic factors (common shrew in river valley and striped field mouse in both habitats), *F* statistic for difference between model including only effect of density-dependent feedback(s) and model with climatic factors

^a^Significance code, 0.001

^b^Significance code, 0.05

^c^Significance code, 0.10

High ambient temperatures in winter, deep snow and high precipitation in spring positively affected population increase rates of the common shrew in meadows, but the combined effect of high precipitation and deep snow had a negative effect on changes in abundance of this species (Table 4). Overall influence of climatic factors on the population increase rate of the root vole in meadows was significant, but effects of single variables were not different from zero (Table 4). Autumn-to-autumn changes of abundance in root voles inhabiting the river valley and striped field mice in both habitats were not significantly affected by climatic factors (Table 4).

## Discussion

The existence of vole cycles has already been demonstrated from Central and Western Europe by, e.g. Tkadlec and Stenseth (2001) and Lambin et al. (2000, 2006), yet the results of these studies were limited to the common vole and the field vole. Here, we provide evidence that oscillating dynamics of other small mammal species can be also driven by a delayed density-dependent process. In the marginal grasslands located within the Białowieża Forest, the population dynamics of all studied species of small mammals was dominated by direct density-dependent feedback, yet the root vole inhabiting meadows and the river valley, and the common shrew in meadows also showed significant delayed density-dependent effects.

The population dynamics of root voles have been studied for almost half a century, but our data are the longest time-series collected for this species so far. After a period of intensive studies (e.g. Lavrova et al. 1960; Tast 1966; Buchalczyk and Pucek 1968; Litvin 1975; Shilov et al. 1977), which suggested a periodical character of abundance changes in this species, the available data on population dynamics of root vole became, with some exceptions (see Ims and Andreassen 2000), very limited.

Our findings confirmed that population cycles of small mammals are not a unique phenomenon restricted to the boreal zone of Eurasia and North America but are fairly widespread (Jędrzejewski and Jędrzejewska 1996). In our study system, population cycles of small mammals occur over a very small spatial scale, whereas earlier reported periodical changes of rodent abundances concerned vast areas of agricultural landscape in the temperate zone (Tkadlec and Stenseth 2001) or open tundra in the arctic zone (Korpimäki and Krebs 1996). In this sense, the population dynamics of root voles in the Białowieża Primeval Forest are more similar to the cycles of field voles observed in northern England, where they are restricted to small patches of open land (clear-cuts) within extensive woodlands (Lambin et al. 2000).

The population dynamics of small mammals inhabiting narrow belts of open landscape within the Białowieża Forest demonstrated here were very different from the regular changes in density of rodents inhabiting adjacent forests, which are driven by multiannual periodicity of seed production by deciduous trees (Pucek et al. 1993; Jędrzejewska and Jędrzejewski 1998). In this paper, we did not attempt to identify proximate factors responsible for the cyclic nature of changes in abundance of small mammals in open habitats, but the difference in population dynamics between plant-eating voles and seed-eating field striped mice suggests different underlying mechanisms. In various Palaearctic biomes, the mean index of rodent cyclicity positively correlates to the mean standing crop of ground vegetation, suggesting that cycles occur only under conditions of constantly high availability of plant food (Jędrzejewski and Jędrzejewska 1996). Thus, the cyclic population dynamics of root voles, which feeds on different parts of sedge and other plants, is probably due to generally high vegetation biomass in open habitats in the Białowieża Forest. Other factors affect the population dynamics of more granivorous field striped mice. Population dynamics of both voles and mice was dominated by first-order processes, suggesting competition for food or space, or the behavioural responses to predators (Berryman 1999). In the case of root voles, the quantity of food is probably not a limiting factor, but availability of food may limit population growth of seed-eating mice. Significant second-order signal in the dynamics of root voles may be caused by a delayed effect of food quality, e.g. level of secondary compounds such as silica (Massey and Hartley 2006; Massey et al. 2008) or predation. However, available data suggest that the role of predation in generating regular changes in population density of voles is rather limited (Lambin et al. 2000; Oli 2003). In our study area, weasels, the main specialised predators, closely followed availability of voles without distinct time delay (Zub et al. 2008), which would be expected if weasels drive vole cycles (Hanski et al. 1991, 2001). Moreover, root voles exhibited clear cyclic population dynamics despite the close neighbourhood of forest rodent community characterised by different population dynamics and presence of predator community of BPF, which is among the richest in Europe (Jędrzejewska and Jędrzejewski 1998). According to the predation hypothesis (Hanski et al. 1991), such predator communities should stabilize rodent population dynamics by acting as generalist predators. We suppose that weasels and generalist predators, which switch to hunt upon voles at their peak density (Jędrzejewska and Jędrzejewski 1998) are responsible for strong direct density-dependent component of population dynamics, whereas food quality may be responsible for the observed delayed density-dependent process.

Root voles and common shrews showed interspecific synchrony in their population dynamics. We also presented long-term changes in relative abundances of small mammals, but we did not observe changes in the type of dynamics, as has been shown in several other areas (e.g. Bierman et al. 2006). The time shift between the peak of root vole population cycle with peak abundance generally being 1 year after the maximum abundance of field mice suggests interspecific competition between these species. As proposed by Gliwicz and Jancewicz (2004), the dominance of root vole in the rodent community results in low densities of other species, when vole population increases.

So far, periodic oscillations of common shrew populations were only reported for Central Siberia (Sheftel 1989), whereas in Fennoscandia, analyses of long-term population dynamics of this species revealed no regular multiannual cycles (Henttonen et al. 1989). Thus, 3-year cycles of common shrews detected by us in the narrow strips of open habitats within a very large temperate woodland is an interesting novel finding. One possible hypothesis explaining the cyclic population dynamics of the common shrew in BPF is that it is a side effect of the cyclic nature of the vole population. The shrew population was able to increase when released from the pressure of predators when voles were abundant. A similar mechanism was suggested already by Henttonen (1985), using a 15-year data set from Fennoscandia. However, data from Fennoscandia demonstrated only the synchrony of low-density phases of voles and shrews, whereas peaks of densities were usually asynchronous (Korpimäki 1986; Sonerud 1988; Henttonen et al. 1989). The synchrony between herbivores and an insectivore is in line with predictions invoking predation as a proximate cause of population crashes, but in BPF, predation impact on shrews and rodents alike is mainly exerted by generalist predators (see Jędrzejewska and Jędrzejewski 1998).

One can expect that if predation explains cyclic dynamics of shrews, it should also affect dynamics of field striped mice. As opposed to shrews, dietary niches of voles and mice partially overlap, especially in spring when field mice often feed on the green parts of plants (Pucek 1984). Mice and shrews differ also in respect to the vulnerability to predation. Striped field mice are more agile and relatively difficult to capture, particularly for weasels, which are the main predator of small mammals in the open areas (Jędrzejewska and Jędrzejewski 1998). An alternative explanation for the discrepancy between cyclic dynamics of shrews and mice is that dynamics of shrews may be indirectly affected by a delayed increase of secondary compounds level, such as silica, in plants. Direct effect of the secondary compounds may drive vole population cycles (Massey et al. 2008), but also may affect plant-eating invertebrates, which are important food for shrews (Churchfield and Rychlik 2006). In the Białowieża Forest, the between-year variation in the common shrew numbers was related to the abundance of forest floor invertebrates (Jędrzejewska and Jędrzejewski 1998).

Climatic factors acting in winter and spring (affecting mainly survival and initial reproduction rates of small mammals) were more important than those acting in summer and autumn. Our results showed that the common shrew was most vulnerable to influences of weather. These animals are characterized by extremely high metabolic rates and live close to their physiological limits, thus they are very sensitive to unfavourable ambient conditions (Ochocińska and Taylor 2005). Interaction between deep snow and high rainfall had a negative effect on population growth of the common shrew in meadows. This combination of weather conditions probably causes flooding of undergrowth nests and could directly affect survival of shrews, increasing their energy expenditures (Hayes and O'Connor 1999). On the other hand, high ambient temperatures in winter and snow cover in spring were positively related to autumn-to-autumn changes in abundance of common shrew. Deep snow provides protection both against low temperatures and predators, especially birds of prey, limiting their access to animals active under the snow (e.g. Jędrzejewski et al. 1994).

In our study, a long-term trend in small mammal abundance was not observed in species typical of open habitats, such as the root vole or harvest mouse, but in species more related to forest habitats, such as the yellow-necked mouse. These directional changes may reflect alteration in habitat caused mainly by secondary forest succession.

According to the available data, two opposite gradients of rodent fluctuations have been found in Europe. In Fennoscandia, the amplitude and periodicity of the cycles increase with latitude, whereas in central Europe, cyclicity increases with decreasing latitude (Tkadlec and Stenseth 2001). Here, we add new evidence that the pattern of small mammal cycles in continental Europe is not necessarily related to latitudinal gradient. Both indicators of cyclicity for small mammal populations in open meadows in Białowieża Primeval Forest, the amplitude of oscillations and the *s*-index, were higher than expected for this latitude (see Tkadlec and Stenseth 2001), which is in line with type of population dynamics predicted for similar habitat types and ground vegetation biomass (Jędrzejewski and Jędrzejewska 1996).

## References

[CR1] Batzli GO (1992) Dynamics of small mammal populations: a review. In: McCullough DR, Barrett RH (eds) Wildlife 2001: Populations. Elsevier Applied Science, New York, pp 831–850

[CR2] Berryman AA (1999). Principles of population dynamics and their application.

[CR3] Berryman AA, Berryman AA (2002). Population cycles: causes and analysis. Population cycles: the case for trophic interactions.

[CR4] Berryman AA, Turchin P (2001) Identifying the density-dependent structure underlying ecological time series. Oikos 92:265–270

[CR5] Berryman AA, Lima M (2007) Detecting the order of population dynamics from time series: Nonlinearity causes spurious diagnosis. Ecology 88:2121–2123 10.1890/06-0609.1 17824443

[CR6] Bierman SM, Fairbairn JP, Petty SJ, Elston DA, Tidhar D, Lambin X (2006). Changes over time in the spatiotemporal dynamics of cyclic populations of field voles (*Microtus agrestis* L.). Am Nat.

[CR7] Bjørnstad ON, Champely S, Stenseth NC, Saitoh T (1996). Cyclicity and stability of grey-sided voles, *Clethrionomys rufocanus*, of Hokkaido: spectral and principal components analyses. Phil Trans R Soc London Biol.

[CR8] Boonstra R, Krebs CJ, Stenseth NC (1998). Population cycles in small mammals: the problem of explaining the low phase. Ecology.

[CR9] Buchalczyk T, Pucek Z (1968). Estimation of the numbers of *Microtus oeconomus* using the standard minimum method. Acta Theriol.

[CR10] Burnham KP, Anderson DR (2002). Model selection and inference: a practical information-theoretic approach.

[CR11] Churchfield S, Rychlik L (2006). Diets and coexistence in *Neomys* and *Sorex* shrews in Białowieża forest, eastern Poland. J Zool.

[CR12] Elton CS (1924). Periodic fluctuations in the numbers of animals: their causes and effects. J Exp Biol.

[CR13] Erlinge S, Hasselquist D, Svensson M, Frodin P, Nilsson P (2000). Reproductive behaviour of female Siberian lemmings during the increase and peak phase of the lemming cycle. Oecologia.

[CR14] Falińska K (2003). Alternative pathways of succession: species turnover patterns in meadows abandoned for 30 years. Phytocoenosis NS 15. Archivum Geobotanicum.

[CR15] Gilg O (2002). The summer decline of the collared lemming, *Dicrostonyx groenlandicus*, in high arctic Greenland. Oikos.

[CR16] Gliwicz J, Jancewicz E, Jędrzejewska B, Wójcik JM (2004). Voles in river valleys. Essays on mammals of Białowieża Forest.

[CR17] Hanski I, Hansson L, Henttonen H (1991). Specialist predators, generalist predators, and the microtine rodent cycle. J Anim Ecol.

[CR18] Hanski I, Henttonen H, Korpimäki E, Oksanen L, Turchin P (2001). Small-rodent dynamics and predation. Ecology.

[CR19] Hayes JP, O'Connor CS (1999). Natural selection on thermogenic capacity of high-altitude deer mice. Evolution.

[CR20] Henttonen H (1985). Predation causing extended low densities in microtine cycles: further evidence from shrew dynamics. Oikos.

[CR21] Henttonen H, McGuire AD, Hansson L (1985). Comparisons of amplitudes and frequencies (spectral analyses) of density variations in long-term data sets of *Clethrionomys* species. Ann Zool Fennici.

[CR22] Henttonen H, Haukisalmi V, Kaikusalo A, Korpimäki E, Norrdahl K, Skarén UAP (1989). Long-term population dynamics of the common shrew *Sorex araneus* in Finland. Ann Zool Fennici.

[CR23] Ims RA, Andreassen HP (2000). Spatial synchronization of vole population dynamics by predatory birds. Nature.

[CR24] Ims RA, Henden J-A, Killengreen ST (2008). Collapsing population cycles. Trends Ecol Evol.

[CR25] Jędrzejewska B, Jędrzejewski W (1998). Predation in vertebrate communities. The Białowieża Primeval Forest as a case study.

[CR26] Jędrzejewski W, Jędrzejewska B (1996). Rodent cycles in relation to biomass and productivity of ground vegetation and predation in the Palearctic. Acta Theriol.

[CR27] Jędrzejewski W, Jędrzejewska B, Zub K, Ruprecht AL, Bystrowski C (1994). Resource use by Tawny owls *Strix aluco* in relation to rodent fluctuations in Białowieża National Park, Poland. J Avian Biol.

[CR28] Klemola T, Pettersen T, Stenseth NC (2003). Trophic interactions in population cycles of voles and lemmings: a model-based synthesis. Adv Ecol Res.

[CR29] Korpimäki E (1986). Predation causing synchronous decline phases in microtine and shrew populations in Western Finland. Oikos.

[CR30] Korpimäki E, Krebs CJ (1996). Predation and population cycles of small mammals—a reassessment of the predation hypothesis. Bioscience.

[CR31] Korpimäki E, Brown PR, Jacob J, Pech RP (2004). The puzzles of population cycles and outbreaks of small mammals solved?. Bioscience.

[CR32] Korslund L, Steen H (2006). Small rodent winter survival: snow conditions limit access to food resources. J Anim Ecol.

[CR33] Krebs CJ, Boutin S, Boonstra R, Sinclair ARE, Smith JNM, Dale MRT, Martin K, Turkington R (1995). Impact of food and predation on the snowshoe hare cycle. Science.

[CR34] Lambin X, Petty SJ, MacKinnon JL (2000). Cyclic dynamics in field vole populations and generalist predation. J Anim Ecol.

[CR35] Lambin X, Bretagnolle V, Yoccoz NG (2006). Vole population cycles in northern and southern Europe: is there a need for different explanations for single pattern?. J Anim Ecol.

[CR36] Lavrova MJ, Prokhorova EV, List LV (1960). Some features of the biology of *Microtus oeconomus* Pall. and the course of Leptospirosis epizootic among the latter in the western part of the Moscow region. Byulleten Moskovskogo Obshschestva Ispytateleï Prirody (Otdel Biologicheskiï).

[CR37] Litvin VY (1975). Materials of the ecology of the tundra vole (*Microtus oeconomus*) obtained by marking method. Fauna and Ecol of the Rodents.

[CR38] Massey FP, Hartley SE (2006). Experimental demonstration of the antiherbivore effects of silica in grasses: impact on foliage digestibility and vole growth rates. Proc R Soc B.

[CR39] Massey FP, Smith MJ, Lambin X, Hartley SE (2008). Are silica defenses in grasses driving vole population cycles?. Biol Lett.

[CR40] Ochocińska D, Taylor JRE (2005). Living at the physiological limits: field and maximum metabolic rates of the common shrew (*Sorex araneus*). Physiol Biochem Zool.

[CR41] Oli MK (2003). Population cycles of small rodents are caused by specialist predators: or are they?. Trends Ecol Evol.

[CR42] Pucek Z (1984). Key for identification of Polish mammals.

[CR43] Pucek Z, Jędrzejewski W, Jędrzejewska B, Pucek M (1993). Rodent population dynamics in a primeval deciduous forest (Białowieża National Park) in relation to weather, seed crop, and predation. Acta Theriol.

[CR44] R Development Core Team (2011). R: A language and environment for statistical computing.

[CR45] Royama T (1992). Analytical population dynamics.

[CR46] Saitoh T (1987). A time-series and geographical analysis of population-dynamics of the red-backed vole in Hokkaido, Japan. Oecologia.

[CR47] Sheftel BI (1989). Long-term and seasonal dynamics of shrews in Central Siberia. Ann Zool Fennici.

[CR48] Shilov IA, Kaletskaya ML, Ivashkina IN, Soldatova AN (1977). The dynamics of the abundance of *Microtus oeconomus* Pall. in the Darwin Nature Reserve. Byulleten Moskovskogo Obshschestva Ispytateleï Prirody (Otdel Biologicheskiï).

[CR49] Sinclair ARE, Olsen PD, Redhead TD (1990). Can predators regulate small mammal populations—evidence from house mouse outbreaks in Australia. Oikos.

[CR50] Singleton G, Krebs CJ, Davis S, Chambers L, Brown P (2001). Reproductive changes in fluctuating house mouse populations in southeastern Australia. Proc R Soc Lond Biol.

[CR51] Smith MJ, White A, Lambin X, Sherratt JA, Begon M (2006). Delayed density-dependent season length alone can lead to rodent population cycles. Am Nat.

[CR52] Sonerud G (1988). What causes extended lows in microtine cycles? Analysis of fluctuations in sympatric shrew and microtine populations in Fennoscandia. Oecologia.

[CR53] Stenseth NC (1999). Population cycles in voles and lemmings: density dependence and phase dependence in a stochastic world. Oikos.

[CR54] Tast J (1966). The root vole, *Microtus oeconomus* (Pallas) as an inhabitant of seasonally flooded land. Ann Zool Fennici.

[CR55] Tkadlec E, Stenseth NC (2001). A new geographical gradient in vole population dynamics. Proc R Soc Lond Biol.

[CR56] Wijnhoven S, Van Der Velde G, Leuven RSEW, Smits AJM (2005). Flooding ecology of voles, mice and shrews: the importance of geomorphological and vegetational heterogeneity in river floodplains. Acta Theriol.

[CR57] Zub K, Sönnichsen L, Szafrańska PA (2008). Habitat requirements of weasels *Mustela nivalis* constrain their impact on prey populations in complex ecosystems of the temperate zone. Oecologia.

